# Wearable teleoperation controller with 2-DoF robotic arm and haptic feedback for enhanced interaction in virtual reality

**DOI:** 10.3389/fnbot.2023.1228587

**Published:** 2023-08-07

**Authors:** Zheyuan Zhang, Chen Qian

**Affiliations:** ^1^The Laboratory for Computational Sensing and Robotics, Department of Mechanical Engineering, Johns Hopkins University, Baltimore, MD, United States; ^2^Department of Mechanical Engineering, University of Michigan, Ann Arbor, MI, United States

**Keywords:** haptics, multimodal fusion, teleoperation, virtual environment, human-robot collaboration

## Abstract

**Introduction:**

Teleoperation is an essential component in fields such as medicine and manufacturing, enabling human operators to interact with remote robotic systems. A wearable device has been designed and manufactured to enhance sensitivity, wearability, and synchronization, providing users with the ability to experience the sensation of grasping virtual objects.

**Methods:**

The device incorporates a 2-DoF robotic arm, haptic sensors for finger gestures, and a Unity-powered virtual scene system. Its effectiveness was evaluated through user experiments, where participants were asked to rank the weights of three virtual balls and identify the direction of force applied to a virtual ball in separate tests. Additionally, the device's ability to render various shapes was also examined.

**Results:**

The experiments showed that 73.3% of participants accurately ranked the balls by mass, and an overall correctness rate of 87.3% was achieved for force direction identification. For shape rendering, the device yielded more accurate results for simple objects like spheres, whereas rendering more complex objects such as cups and cones was challenging.

**Discussion:**

The findings indicate that this wearable device has potential applications in haptic feedback and virtual reality contexts. Despite the challenges with complex shape rendering, the device shows promising capability in enhancing the user's immersive experience in virtual environments.

## 1. Introduction

Teleoperation is a process where human operators form part of the control loop of robotic systems, allowing for human-based high-level planning and cognitive decisions, while robots handle mechanical operations. This technology offers significant potential in the medical field, enabling remote control of surgical equipment and thereby minimizing exposure to contamination or infectious agents. One manifestation of this technology is the Tele-Robotic Intelligent Nursing Assistant (TRINA; Li et al., 2017), developed by researchers at Duke University. TRINA has been designed to assist healthcare workers in routine patient care tasks and manage contaminated materials and protective gear. The system is comprised of robotic arms for operation and remote haptic devices for control, and it has been successful in performing 60% of nursing tasks (Li et al., 2017).

The role of multimodal fusion is critical in enhancing human-computer interaction (Ning et al., 2023). This process involves combining data from multiple sources to deliver a more seamless and intuitive user experience (Meng and Song, 2020). In the research concerning the haptic device, an investigation is underway to ascertain the potential benefits of integrating haptic feedback with other sensory modalities, such as vision and auditory cues. The incorporation of haptic information into a multimodal fusion framework aims to foster a more comprehensive understanding of the environment for the user. This should improve their ability to perform tasks and interact with the system effectively. This approach is in line with the growing trend in human-computer interaction research, where efforts are being made to develop more natural and efficient methods for human-technology engagement using a combination of sensory inputs.

Haptic VR controllers play a crucial role in interacting with virtual content and are available in various shapes and functions, from handheld controllers to haptic gloves (Inc., 2020). Most commercial devices offer only vibrotactile feedback (Maereg et al., 2017), but researchers have developed a wide variety of handheld controllers rendering texture, shape, grasp, squeeze feedback, shifting weight (Choi et al., 2017; Jin et al., 2017), and haptic behavior for two-handed use (Murayama et al., 2004). The primary limitation of these devices is their continuous contact with the user's hand, which may undermine the sensation of free-hand interactions and require occasional placement aside for physical-world hand usage.

Haptic VR controllers are key in interaction with virtual content. Available in various forms and functions, these range from handheld controllers to haptic gloves. Although most commercial devices offer only vibrotactile feedback, a wide variety of handheld controllers rendering texture, shape, grasp, squeeze feedback, shifting weight, and haptic behavior for two-handed use, have been developed by researchers. A primary limitation of these devices is their continuous contact with the user's hand, which could detract from the sensation of free-hand interactions and require occasional placement aside for physical-world hand usage. Physical proxies, or encounter-type haptics, are another approach for creating haptic sensations in virtual environments (Zhu et al., 2019). However, this method either limits scripted experiences or necessitates expensive and large machinery for dynamic positioning, such as robotic arms (Araujo et al., 2016), moving platforms (Jin et al., 2021), or multiple human helpers. While the fidelity of the provided haptic sensation is high, limitations include the workspace and actuation speed.

PIVOT (Kovacs et al., 2020) is a wrist-worn haptic device that overcomes some traditional haptic shortcomings by rendering virtual objects into the users' hand on demand. PIVOT is defined as a 1 degree-of-freedom (DoF) wearable-encounter-type haptic device and contacts the users' palm only when required (Kovacs et al., 2020), ensuring the full range of motion and dexterity of users' hands when the device is not in use. However, there are two problems due to its 1-DoF structure, including limitations in rendering force inertia and force from different directions.

Inspired by TRINA and PIVOT, we aim to develop a teleoperation controller system utilizing a 2-DoF robotic arm. Our device seeks to combine the versatility of handheld haptic devices with the high realism of physical agents. Our device enables users to interact with objects in virtual reality while providing haptic force feedback. By incorporating a shape rendering system inspired by Stanford University's Wolverine research (Choi et al., 2016), we enhance the user's perception of the interaction between the gripped object and the controller, making control more intuitive. Furthermore, such kind of systems could be applied on “warehouse, social service, and manufacturing" (Nemlekar et al., 2019).

**Figure 1:**
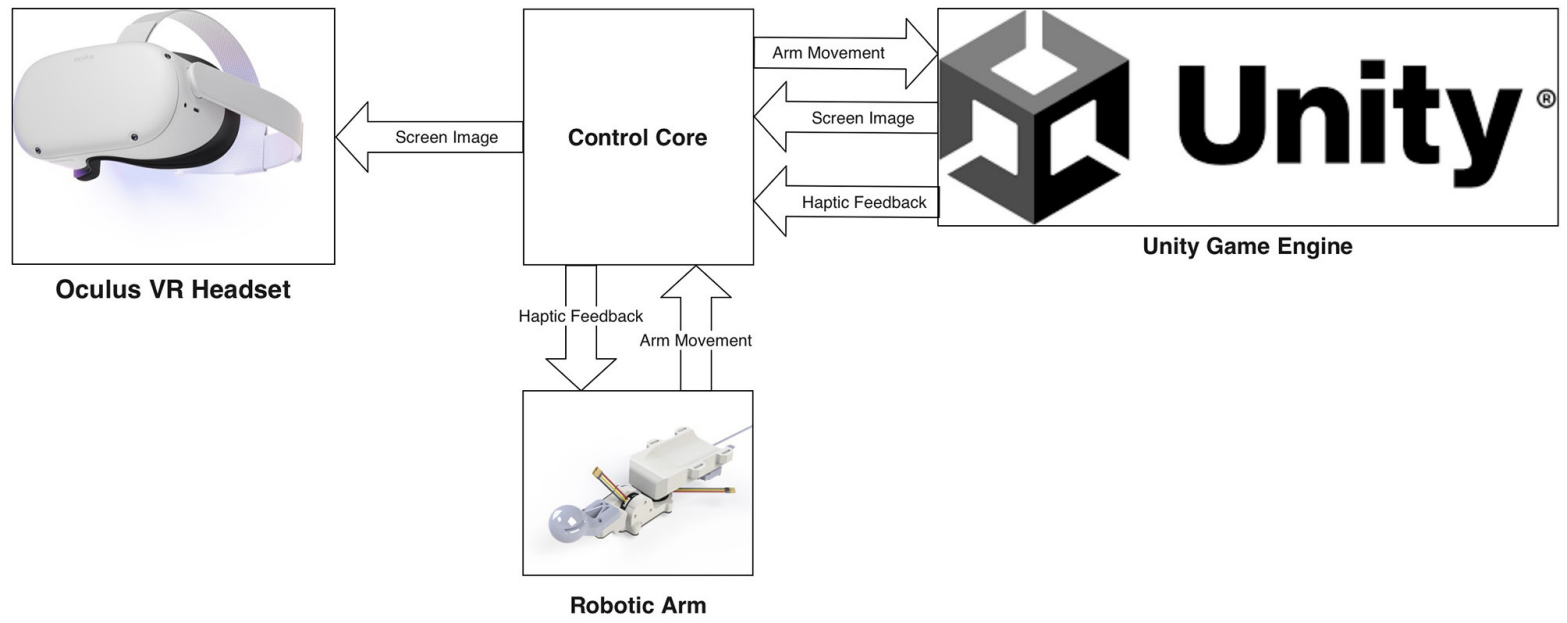
The haptic device and VR system framework (Unity Technologies, 2021; Meta Platforms Inc., 2023).

Our design consists of three interconnected modules: a 2-DoF robotic arm, haptic sensors for finger gestures, and a virtual scene system powered by Unity, which was shown in Figure 1.

The robotic arm is attached to the user's forearm, with a handle in the palm and small tactile sensors attached to the fingers. The VR headset's camera captures arm and wrist movements while haptic sensors detect finger gestures (Ning, 2023). These movements are parameterized into the control core and translated into images in the virtual environment.

In the virtual environment, interactions are initiated as the arm, designed to replicate user's hand gestures, engages with virtual entities. These interactions are meticulously captured, and a sense of shape and weight is communicated to the user through the strategic control of the robotic arm and tactile sensors. Meanwhile, the accompanying head device renders the virtual scene, facilitating an immersive experience. This methodology surpasses the capabilities of conventional feedback-free controller operations, aspiring to deliver a lifelike user experience.

Contextualizing this advancement necessitates a comparison with traditional robotic systems. Crucial references include studies such as: “Implementation of ANN-based auto-adjustable for a pneumatic servo system embedded on FPGA” (Cabrera-Rufino et al., 2022); “An FPGA-based open architecture industrial robot controller” Martínez-Prado et al. (2018); “A multidisciplinary industrial robot approach for teaching mechatronics-related courses” (Garduño-Aparicio et al., 2018); “A PID-type fuzzy logic controller-based approach for motion control applications” (García-Martínez et al., 2020); and “Concurrent optimization for selection and control of AC servomotors on the powertrain of industrial robots” (Padilla-Garcia et al., 2018). These studies offer profound insights into the evolutionary trajectory of robotics, illustrating the gradual steps taken toward a more immersive and realistic user experience. This backdrop of knowledge underscores the significant progress encapsulated in the haptic system presented in this manuscript.

The organization of this study is described as follows: Section 1 addresses the key problem or controversy under investigation, namely the deficits or gaps in current haptic technology compared to earlier works. It discusses the limitations of existing devices and the potential advancements made possible through this work. The subsequent sections detail the study's methodology, experimental design, results, and interpretation. The methods section outlines the design and development of the 2-DoF robotic arm and the integration of haptic sensors and a Unity-powered virtual scene system. The experimental design section explains the procedure and conditions under which the device was tested. The results section presents the device's performance in terms of weight perception accuracy, force direction identification, and shape rendering. Finally, the interpretation section discusses the implications of these results in the broader context of haptic feedback and virtual reality applications.

The most significant findings or main contributions of this work revolve around the creation and testing of a wearable haptic device capable of providing a realistic experience of interaction with virtual objects. Despite some limitations in accurately rendering complex shapes, the device demonstrated a high degree of success in tasks involving weight perception and force direction identification. These results indicate that the device holds considerable potential for various applications, which could transform the way users interact with technology and virtual reality environments. This study presents a novel advancement in the field of teleoperation and haptic feedback technology, offering a more intuitive and realistic user experience.

## 2. Materials and methods

This section describes the methods employed in the present study, aimed at evaluating the efficacy of the proposed hybrid haptic controller. The materials used for the development of the system, the participants involved in the user study, and the procedures followed during the study are detailed. Furthermore, the methods for threshold identification, haptic device iteration, and immersive virtual environment and intelligent grabber recognition are explained. The design of the haptic force rendering control, the statics calculation and tolerance analysis, and the experimental validation and data analysis processes are also elaborated.

### 2.1. Participants

In the study, 15 participants from the Zhejiang University—University of Illinois Urbana-Champaign Institute (11 males, four females, average age 22.4) with no prior haptic device usage experience took part. All participants provided informed consent and participated in a single experimental session. While it might be beneficial to conduct multiple sessions to further understand the learning effect and longer-term usability, a single session was chosen for the initial assessment to evaluate the immediate usability and effectiveness of the device, as well as to accommodate participant availability and the study's timeline.

A diverse group of participants was included in this study, among which six had prior experience with VR headsets or similar devices. For the experiment, each participant's right hand was fitted with the haptic device and adjustments were made using the device's retractable structure to ensure a comfortable fit. Participants were thoroughly briefed on the nature and proceedings of the experiment, along with the implementation of strict safety measures. These included the setting of speed and force limits for the robotic arm and provision of an on/off stop device controlled by the experimenter. Participants were also informed about their right to withdraw from the experiment at any time without needing to provide a reason. The study was conducted under a joint collaboration between Zhejiang University and the University of Illinois Urbana-Champaign Institute and received approval from the institutional review board at Zhejiang University. While the first experiment had 15 participants, the second and third experiments saw a reduced number of participants with 11 and 10 individuals respectively, ensuring the inclusion of the six participants with prior VR experience in all experiments.

### 2.2. Procedure

The objective of this study is to develop a novel hybrid controller integrating a 2-DoF robotic arm, haptic sensors for finger gestures, and a Unity-powered virtual scene system (Figure 1). The proposed controller is intended to offer several features: (1) the capacity to grasp and release airborne objects; (2) the delivery of haptic feedback during the act of grasping and releasing stationary items; (3) the simulation of dynamic forces from held objects, such as weight, inertia, or resistance; and (4) the accommodation of unrestricted hand movements as necessary. By affording a more immersive and realistic user experience compared to traditional feedback-free VR controllers, this hybrid controller is expected to facilitate various interactions with virtual objects.

In order to evaluate the efficacy of the proposed hybrid controller, a user study was conducted, consisting of four tasks: (1) grasping balls of the same size but with different weights; (2) grasping and throwing differently shaped objects with varying weights; (3) assessing the effectiveness of force rendering; and (4) evaluating the effectiveness of shape rendering by grasping different objects. Performance of the hybrid controller in these tasks was compared with that of available exoskeleton systems in an effort to comprehend its potential advantages and capabilities.

Through this research, it is hoped to elucidate the potential advantages of the proposed hybrid controller in offering a more realistic and immersive VR experience, which may eventually contribute to the development of more portable and efficient devices for a range of applications.

### 2.3. Materials

The haptic VR system comprises two main subsystems that contribute to a realistic and immersive user experience. The first subsystem is the virtual scene system powered by Unity, which renders the visual representation of the virtual environment and the interactions between the user and virtual objects. The second subsystem is the 2-DoF robotic arm integrated with haptic sensors for finger gestures, responsible for providing haptic feedback that simulates the sensation of grasping and releasing objects in the virtual environment.

The VR environment is created using the Unity game engine, which allows for the design and programming of realistic physics-based interactions between virtual objects and the user's hand gestures. The environment incorporates various types of objects and interactions, including grasping balls of different weights and catching and throwing objects of varying shapes and sizes.

The 2-DoF robotic arm is a custom-designed mechanism attached to the user's forearm, featuring a handle in the palm and small tactile sensors on the fingers. The arm is designed to provide force feedback and simulate various dynamic forces from held objects, such as weight, inertia, and resistance. It also allows for unrestricted hand movements when necessary. The haptic sensors integrated with the robotic arm are responsible for detecting finger gestures and translating them into virtual interactions in the VR environment.

In combination, these subsystems work together to provide a more realistic and immersive experience for users interacting with virtual objects in the VR environment. The performance of this hybrid haptic VR system is evaluated in a user study, which includes tasks designed to assess the effectiveness of force and shape rendering, as well as the overall user experience.

### 2.4. Methods

Understanding the overall structure of the approach before delving into the individual components of the methods is crucial for this study. It strives to develop an innovative haptic device using a multi-faceted approach, focusing on hardware design iterations, an embedded system design, and the creation of an immersive virtual environment.

In the subsequent subsections, each aspect of the methodology is detailed, starting with the process of threshold identification. This process forms the foundation for the dimensions of the CAD model and its simulation. Following this, the iterative hardware design process that leads to the creation of the final haptic device prototype is thoroughly examined.

In the subsequent sections, the design of the embedded system powering the haptic device is explored, ensuring seamless operation and efficient power management. Finally, the immersive virtual environment and intelligent Grabber recognition are explained. These enhancements aim to improve the user experience by facilitating intuitive interaction with virtual objects. This comprehensive overview is designed to provide readers with a thorough understanding of the various elements contributing to the development of the advanced haptic device.

#### 2.4.1. Threshold identification

**Figure 2:**
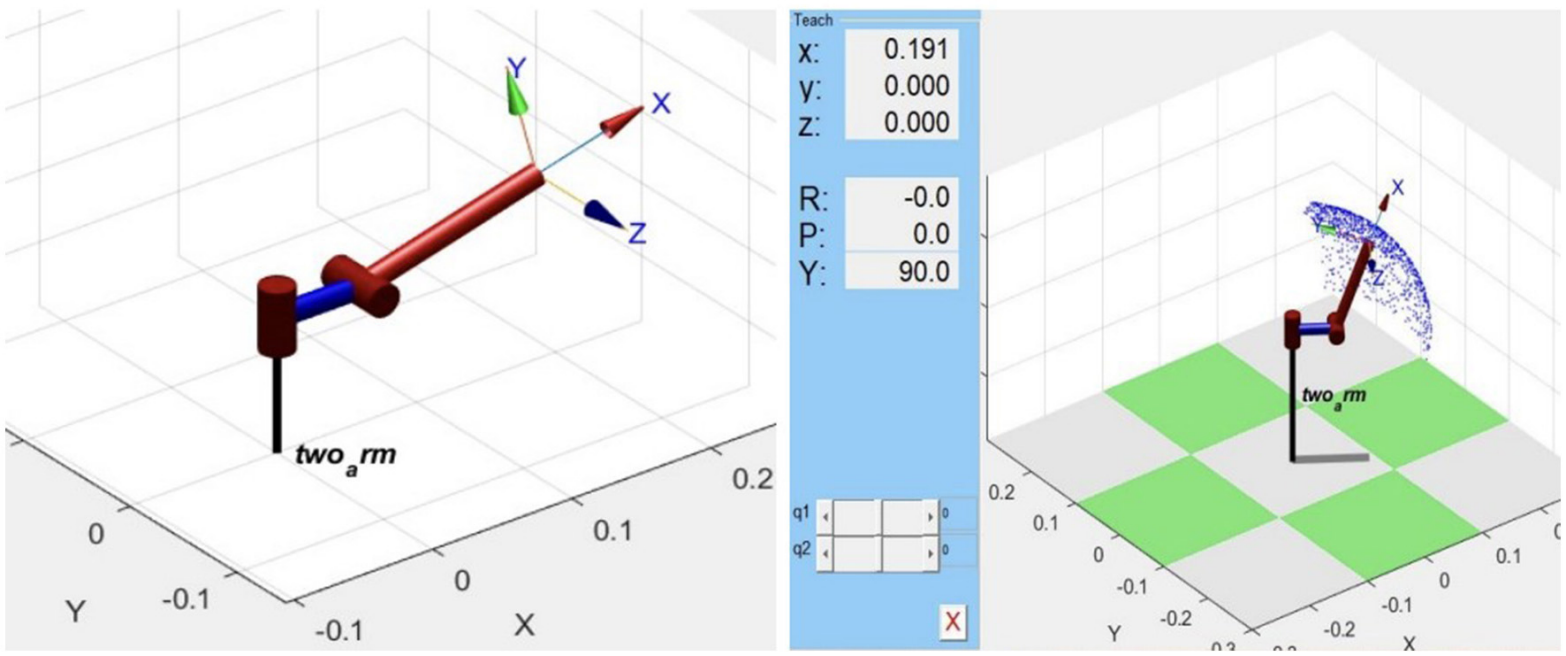
Haptic device simulation and workspace illustration.

Before creating the CAD model in Solidworks software, we must determine the dimensions of the 2-DOF device and simulate its workspace. The dimensions must be such that the workspace covers the positions of one of our hands. Consequently, we first use MATLAB to simulate the model of the 2-DOF device and illustrate its workspace shown in Figure 2.

With the simulation, the yaw and pitch axis rotation ranges are identified as well. The following image shows the required motor rotation range, which is also calculated using MATLAB. In our final design, the rotation range is larger than the requirement, ensuring that it meets the desired specifications.

#### 2.4.2. Haptic device iteration

Our haptic device leverages a blend of a 2-DoF robotic arm powered by Unity and a virtual scene system. The robotic arm is attached to the user's arm, with a handle positioned on the palm and small haptic sensors on the fingers. During operation, the robotic arm captures the movements of the arm and wrist, while the haptic sensors track the gestures of the arm and hand. This motion data is parameterized into the control core, and the robotic arm in the virtual scene mimics these movements to interact with virtual objects.

**Figure 3:**
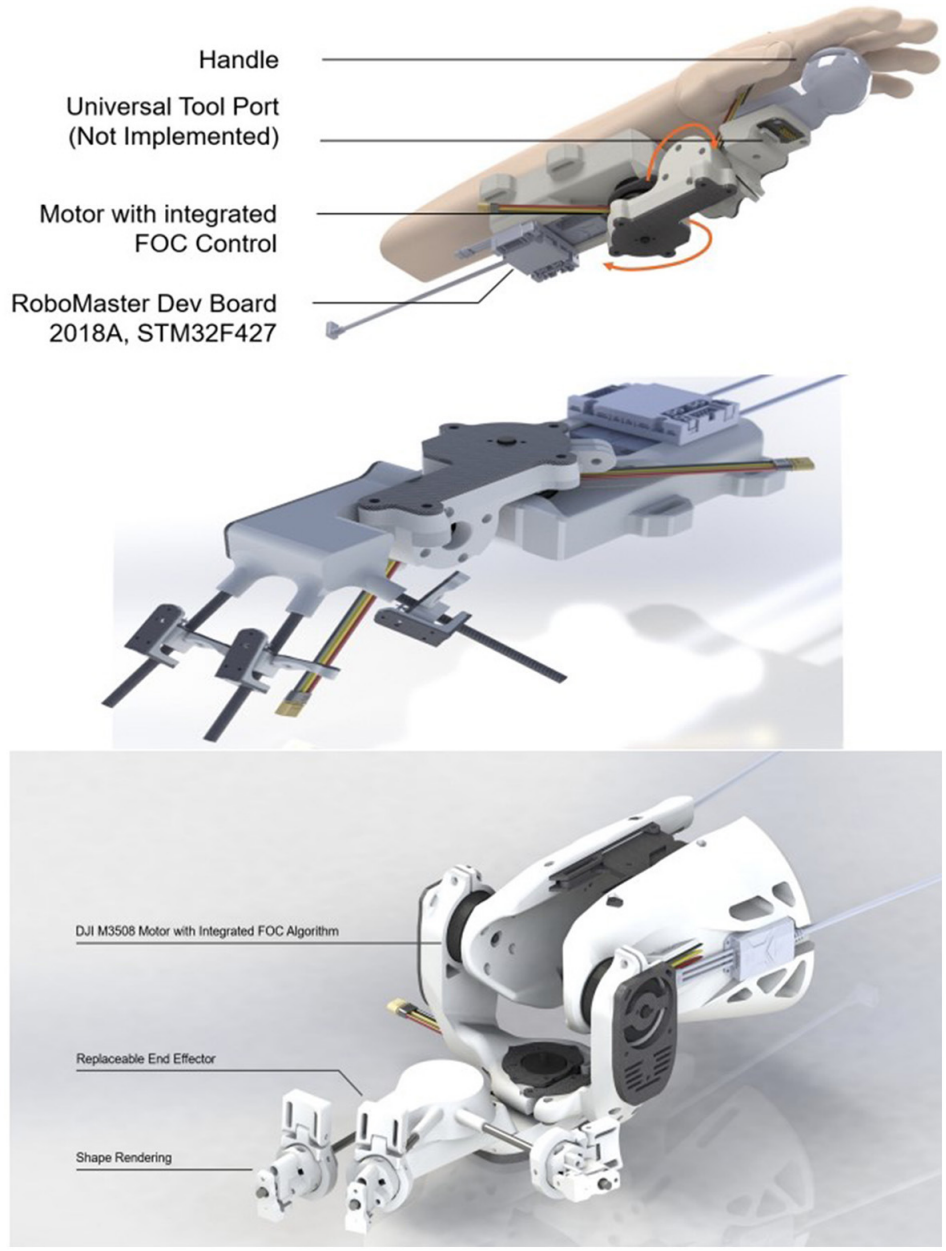
Design of first prototype, second prototype, and final prototype.

The first prototype, depicted in Figure 3, comprises a 2-DoF robotic arm attached to the user's forearm, assembled using 3D printed parts and acrylic panels. The microcontroller STM32F427 is mounted on the first joint and connected to two motors via two ESCs. This initial version demonstrated force rendering through the torque provided by the motors.

We then added shape rendering functionality, leading to the second prototype shown in Figure 3. Inspired by Stanford's haptic device (Choi et al., 2016), we used a small motor to pull a wire attached to a slider, limiting the operation on a carbon tube. With three sliders, the fingers could slide freely on the carbon tube, effectively rendering the shape.

During the testing phase of the second prototype, we identified a potential issue-high torque from the motor could cause overheating and damage. To resolve this, we employed two motors to drive the first joint and adjusted the mechanics accordingly. The final prototype is displayed in Figure 3. In addition to this, we improved the shape rendering design to allow fingers to assume a variety of poses more freely, simulating different shapes.

The iterations of the prototypes were necessary to resolve design issues and enhance functionality, providing more effective force and shape rendering. Each version was crucial in the evolution of the design, leading to the final robust and efficient haptic device that is presented in this study.

**Figure 4:**
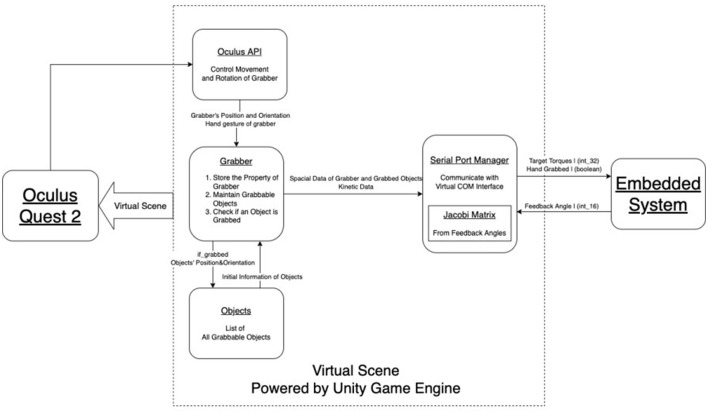
Top-level software block diagram.

Figure 4 presents the block diagram of our innovative software system, highlighting the seamless integration of two primary classes: Grabber and Serial Port Manager. The Grabber class is responsible for managing interactions with objects in the virtual scene, while the Serial Port Manager class handles communication with the embedded system.

This inventive design reflects our commitment to creating a user-friendly and efficient haptic device. By developing distinct classes for each core function, our software system effectively addresses the challenges of virtual object interaction and reliable communication with the embedded system. This creative approach ensures a more immersive and realistic experience for users, ultimately enhancing the overall performance of the haptic device.

#### 2.4.3. Haptic device embedded system design

**Figure 5:**
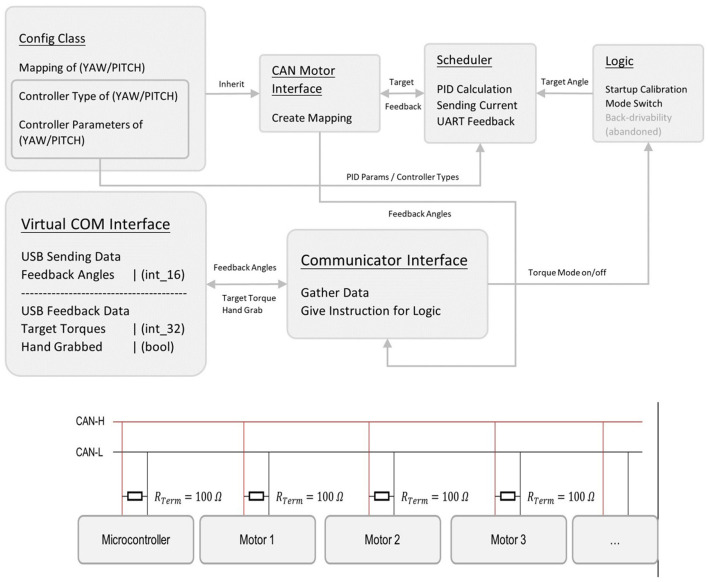
Block diagram of our embedded system (including data transfer direction) and controlled area network (CAN).

Our haptic device features a cutting-edge embedded system design, integrating components such as power supply, embedded module, and communication networks to achieve seamless operation. The block diagram of our embedded system, including data transfer direction, is shown in Figure 5.

The embedded module is powered by a reliable and high-capacity DJI TB-47D battery, which can accommodate a wide range of voltage inputs. The module is comprised of two M3508 motors, their C620 motor controllers, and an STM32F4 micro-controller. This setup allows for precise control of the output torque on joints, as well as the ability to design various end effectors with different sensors or actuators, thanks to a universal port with 12 pins (Figure 5).

For communication purposes, we utilize a controlled area network (CAN-BUS) for motor-to-MCU communication and a virtual COM port for MCU-to-host PC communication. Our embedded system runs on ChibiOS, a real-time operating system, which enables simpler, more encapsulated, and modular code.

Our innovative embedded system design ensures optimal performance, efficient power management, and a user-friendly experience. By utilizing cutting-edge components and software, we have created a haptic device that is both robust and adaptable to a variety of applications.

#### 2.4.4. Immersive virtual environment and intelligent Grabber recognition

The virtual environment is first rendered in Oculus Quest using Unity, and hand tracking is implemented with an inside-out camera on the device. The HandPosing package is used to implement grasping in Unity, where real-time hand poses are compared to pre-stored poses to determine if an object is grasped. Once an object is grasped, its acceleration is calculated using the derivative of its position and filtered through a low-pass filter to reduce noise. This acceleration is then combined with gravity and multiplied by the object's mass to obtain the interaction force between the object and the hand.

**Figure 6:**
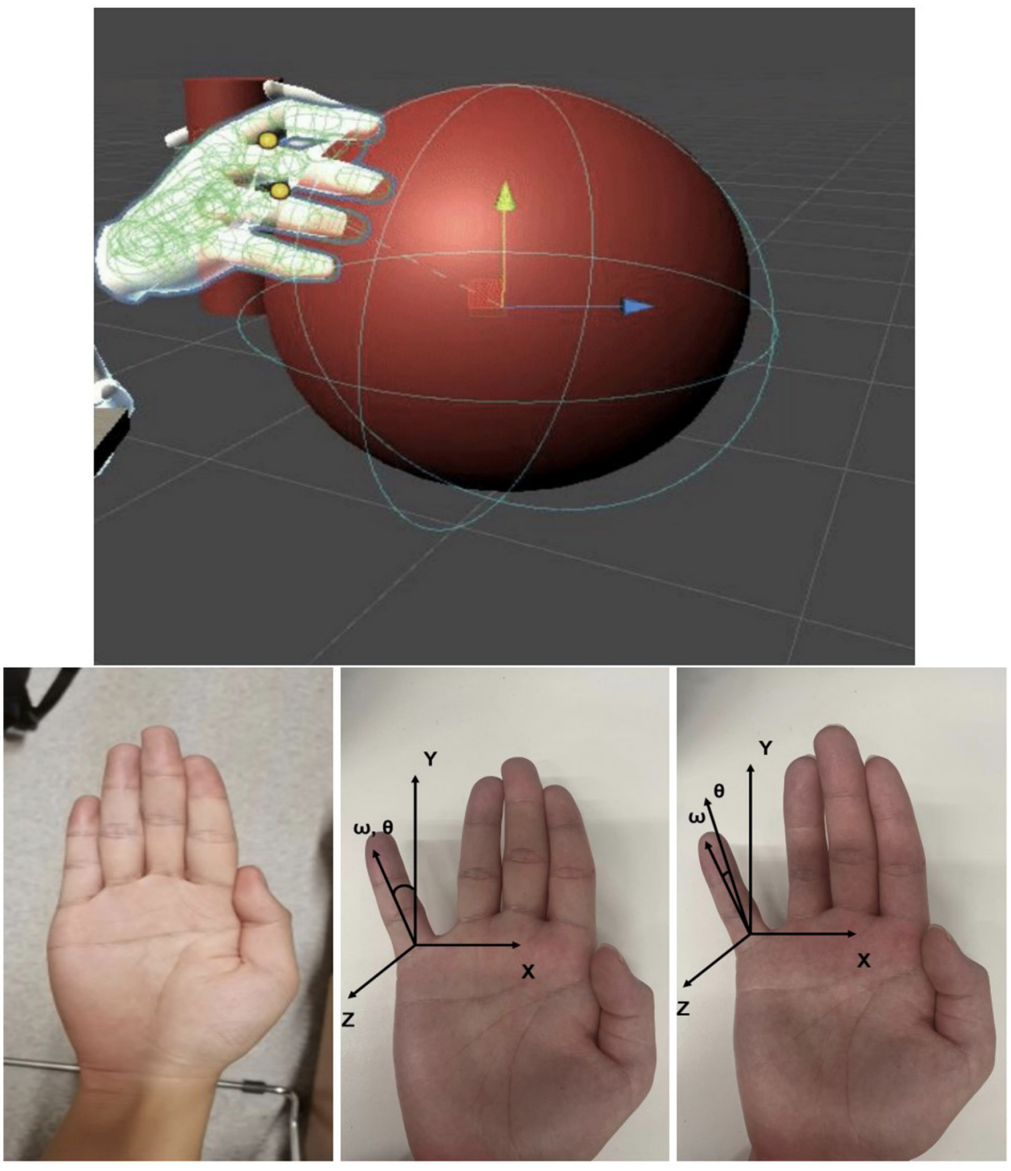
Virtual object and hand gesture recognition.

The core of our software is the innovative Grabber component, designed to facilitate seamless user interaction with virtual objects in an immersive environment. A visual hand model is provided, allowing users to intuitively engage with virtual objects by getting close to the target object and mimicking a preset hand gesture (Figure 6).

Leveraging the Oculus API, our Grabber integrates with Unity and the Oculus Integration library to capture the user's hand movements and spatial information, subsequently rendering the virtual scene in the head display device.

To detect and process grabbing actions, we developed a custom structure for hand gestures, inspired by the natural movement of the human hand (Figure 6). Each hand gesture can be represented by a series of rotation matrices, enabling precise tracking of the virtual hand's movements.

Rotation matrices are used to represent rotations in three-dimensional space. Given a unit vector $\vec{n}=\left(n_{x},n_{y},n_{z}\right)$representing the rotation axis and an angle θ representing the rotation amount, the rotation matrix *R* can be derived as follows:


(1) $$\begin{array}{l} R=[\begin{array}{cc} n_{x}^{2}(1-\cos\theta )+\cos\theta & n_{x}n_{y}(1-\cos\theta )-n_{z}\sin\theta \\ n_{x}n_{y}(1-\cos\theta )+n_{z}\sin\theta & n_{y}^{2}(1-\cos\theta )+\cos\theta \\ n_{x}n_{z}(1-\cos\theta )-n_{y}\sin\theta & n_{y}n_{z}(1-\cos\theta )+n_{x}\sin\theta \end{array}\;\\ \;\begin{array}{c} n_{x}n_{z}(1-\cos\theta )+n_{y}\sin\theta \\ n_{y}n_{z}(1-\cos\theta )-n_{x}\sin\theta \\ n_{z}^{2}(1-\cos\theta )+\cos\theta \end{array}] \end{array}$$

Synchronizing with Unity and Oculus, the virtual hand's gesture structure is compared to the preset gesture structures of virtual objects. Grabbable objects register their necessary information with Grabber upon scene load, including the Hand Gesture structure and initial position and orientation.

The Grabber recognizes grabbing actions by calculating the Norm of differences between Rotation Matrices, comparing internal and preset structures. If the magnitude of the Norm is below a threshold value, the Grabber captures the grabbing event and completes corresponding actions, such as updating the position of the grabbed object and transmitting relevant information to the Serial Port Manager.

Our creative approach to the immersive virtual environment and intelligent Grabber recognition allows for a more natural and intuitive user experience, bridging the gap between the virtual and real world.

### 2.5. Statics calculation and tolerance analysis

In our study, when a user touches an object in the virtual environment and the VR camera detects that the user's hand has grasped the object, we need to simulate the force and inertia at the tip of the device within the virtual environment. To accomplish this, we calculate the corresponding torque of the two motors and control the magnitude using a PID controller. We employ statics calculations to obtain the torque under ideal conditions.

The relationship between the torque and force at the tip of the device is given by the equation:


(2) $$\begin{array}{l} \vec{\tau }=J^{T}\left(\theta \right)*f_{tip} \end{array}$$

In the equations provided, the following variables are used: $\vec{\tau }$: This vector represents the torque applied at each motor of the haptic device. *J*^*T*^(θ): This is the transpose of the Jacobian matrix *J*(θ). The Jacobian matrix, which is a function of the joint angles θ, is crucial for mapping joint velocities to end-effector velocities in a robotic arm or similar manipulator. *f*_*tip*_: This represents the force at the tip of the device.

Using the screw axis, we calculate the body Jacobian matrix, which allows us to determine the torque of each motor through the following expression. The Jacobian matrix for our device is:


(3) $$\begin{array}{l} J(\theta )=\left[\begin{array}{cc} \begin{array}{l} 0\\ sin(\theta_{2})\\ cos(\theta_{2})\\ -0.06cos(\theta_{2}^{2})-0.125cos(\theta_{2})\\ \;+0.03925sin(\theta_{2})-0.06sin(\theta_{2}^{2})\\ 0\\ 0 \end{array} & \begin{array}{l} \;1\\ \;0\\ \;0\\ \;0\\ -0.03925\\ \;0.125 \end{array} \end{array}\right] \end{array}$$

θ_2_ is the joint angles in the manipulator. In the context of the Jacobian matrix, these angles are used to calculate the effect of changing a particular joint angle on the position and orientation of the end effector (the tip of the device). These equations serve the purpose of determining the required motor torques ($\vec{\tau }$) for given forces at the device's tip (*f*_*tip*_), allowing for accurate haptic feedback in the virtual environment. By employing these calculations and equations, our haptic device can accurately simulate the forces and torques required for a realistic interaction within the virtual environment.

Our current design utilizes the cameras on the Oculus Quest 2 VR equipment to recognize a user's hand (Clark and Moodley, 2016). If the visual localization system is not reliable, it may result in a glitchy interaction experience when interacting with the device. As such, the precision of the localization system is critical and warrants testing. During the grabbing process, the end-effector's position should correspond to the geometric center of the user's hand. We have users grasp the end effector while collecting data from the onboard AHRS and motor feedback to calculate the end-effector's position, comparing the calculated position with the position recognized by the visual localization system.

We employ the following formula, which is based on the forward kinematic equation:


(4) $$\begin{array}{l} T\left(\theta \right)=e^{\left[S_{1}\right]\theta_{1}}\cdot e^{\left[S_{2}\right]\theta_{2}}\cdot M \end{array}$$

In the provided equations, the following variables and symbols are used: *T*(θ): This represents the transformation matrix, or pose, of the AHRS (Attitude and Heading Reference System) in the spatial frame. This pose is a function of the joint angles, denoted as θ_1_ and θ_2_. $e^{\left[S_{1}\right]\theta_{1}}$ and $e^{\left[S_{2}\right]\theta_{2}}$: These are exponential transformations based on the screw axis S and joint angle θ. They describe the individual movements at each joint. M: This symbol represents the initial configuration of the manipulator when all the joint angles are zero. It is not directly defined in the explanation given, but this is a standard meaning in robotics literature.

The *T* matrix is 4 × 4, with *T*[0][2], *T*[1][2], *T*[2][2] denoting the *x, y, z* coordinates of the end effector. We integrate the error between the calculated position and the visually observed position using the equation:


(5) $$\begin{array}{l} error_{cumulative}=\int \left(pos_{visual}-pos_{AHRS}\right)dt \end{array}$$

In the provided equations, the following variables and symbols are used: *pos*_*visual*_: This variable represents the position of the end effector as visually observed. *pos*_*AHRS*_: This variable represents the position of the end effector as calculated by the AHRS. *error*_*cumulative*_: This is the cumulative error, calculated as the integral of the difference between the visually observed position and the AHRS calculated position over time.

The magnitude of *error*_*cumulative*_ indicates the reliability of the visual localization system. Test results are listed in Table 1.

**Table 1:** Cumulative difference of visual loc w.r.t AHRS loc (x, y, and z) of each trial.

**Trial #**	**Cumulative difference of visual loc w.r.t AHRS loc (x, y, and z)**	**Duration (s)**
1	(−14 mm, 22 mm, 30 mm)	61.54
2	(−15 mm, −15 mm, 16 mm)	55.23
3	(−4 mm, −17 mm, −12 mm)	77.62
4	(−20 mm, 13 mm, −18 mm)	43.41

From these results, we observe that the cumulative difference between the visual and AHRS locations is relatively small, suggesting that the visual localization system and AHRS conform well. Consequently, the visual localization system should be reliable enough to provide users with a better experience.

### 2.6. Haptic force rendering control design

The low-level controller operates in two distinct modes: torque mode and angle mode, which are determined by input signals from the high-level controller.

In torque mode, the DJI C620 motor controller's internal Field-Oriented Control (FOC) algorithm is utilized to generate motor torque output, simulating the gravity and inertia of objects in a virtual environment. This mode is activated when the high-level controller detects users grabbing objects. Mathematically, the FOC algorithm can be represented as follows:


(6) $$\begin{array}{l} T_{output}=FOC\left(T_{input},\theta_{motor},I_{d},I_{q}\right) \end{array}$$

Where *T*_*output*_ is the motor torque output, *T*_*input*_ is the input torque,θ_*motor*_ is the motor's rotor angle, and *I*_*d*_ and *I*_*q*_ are the direct and quadrature current components, respectively.

Conversely, when the high-level controller identifies that users are not holding objects, angle mode is enabled. Angle mode incorporates a PD/PI controller, which combines Proportional-Derivative (PD) and Proportional-Integral (PI) control methods to effectively manage the joint angle. The PD controller calculates the desired speed from the desired angle, while the PI controller computes the desired torque output from the desired speed. This combination allows for smooth and accurate movement, gradually adjusting the joint angle to align with the user's palm. The PD and PI controllers can be expressed mathematically as:


(7) $$\begin{array}{l} V_{desired}=K_{p}\cdot \left(\theta_{desired}-\theta_{current}\right)-K_{d}\cdot {\dot{\theta }}_{current} \end{array}$$

(8) $$\begin{array}{l} T_{desired}=K_{p^{\prime }}\cdot \left(V_{desired}-V_{current}\right)-K_{i^{\prime }}\int \left(V_{desired}-V_{current}\right)dt \end{array}$$

**Figure 7:**
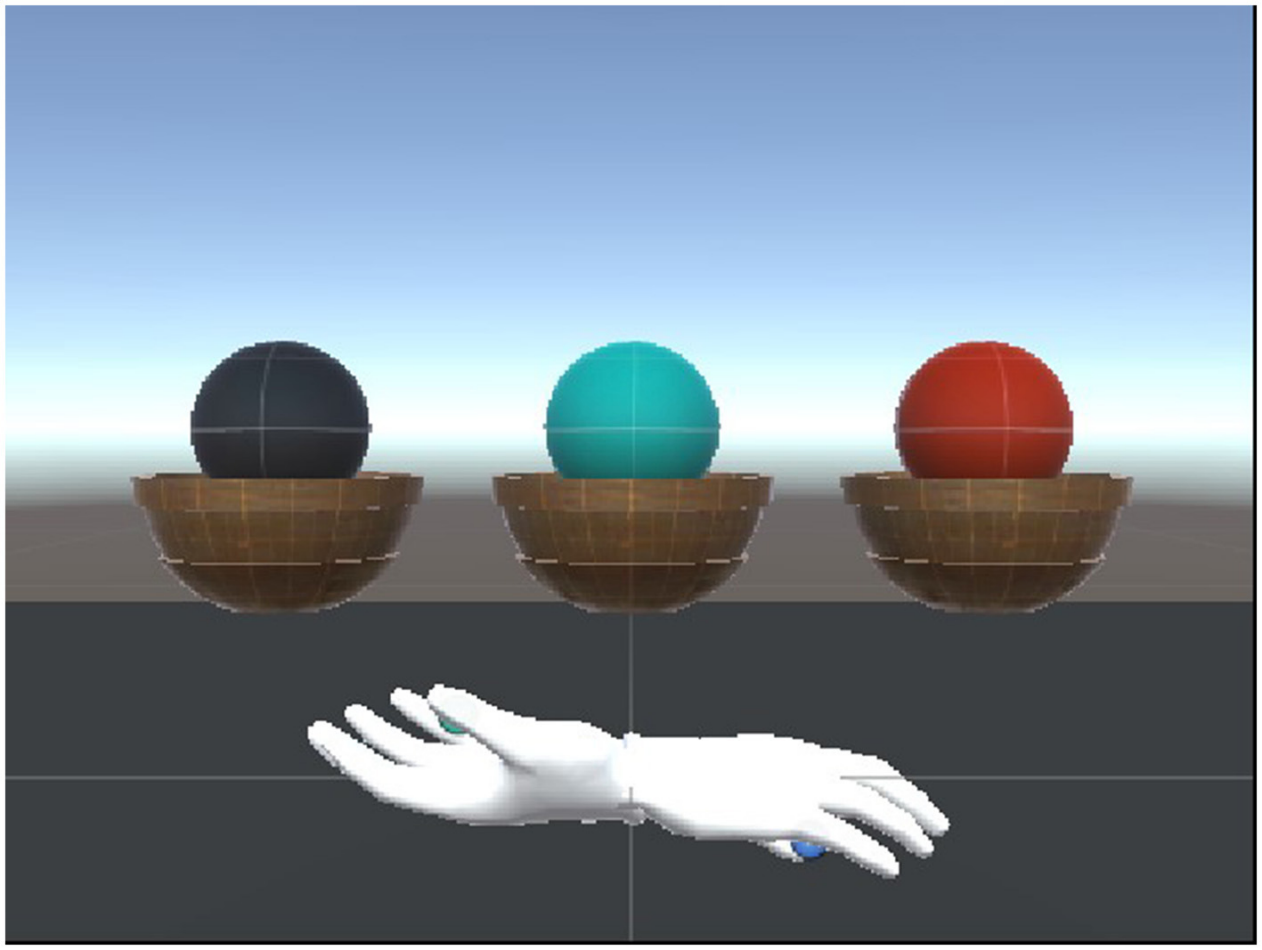
Participants were presented with three virtual balls (1,000, 2,000, and 3,000 g) and asked to estimate their weights and rank them from heaviest to lightest.

Where *V*_*desired*_ and *V*_*current*_ are the desired and current joint speeds, θ_*desired*_ and θ_*current*_ are the desired and current joint angles, *K*_*p*_, *K*_*d*_, $K_{p^{\prime }}$, and $K_{i^{\prime }}$ are the controller gains. Figure 7 shows a brief structure of the embedded system.

### 2.7. Experimental validation and data analysis

The following sections present a detailed explanation of the methods used to evaluate the functionality and realism of the haptic device. The assessment is divided into three subsections, focusing on weight perception, force rendering effectiveness, and the capability of simulating different shapes while maintaining constant weight.

#### 2.7.1. Assessing weight perception

To assess the realism of our rendering device, 15 participants were tasked with ranking the weights of three virtual balls of equal size but varying weights. Three differently colored virtual spheres, each with distinct masses, were presented within the scene. The balls had masses of 1,000, 2,000, and 3,000 g, and were scaled by a factor of 6 to ensure that the output torque on each motor did not surpass the designated threshold. Participants were guided to grasp the virtual balls several times and rank their perceived weights from heaviest to lightest.

**Figure 8:**
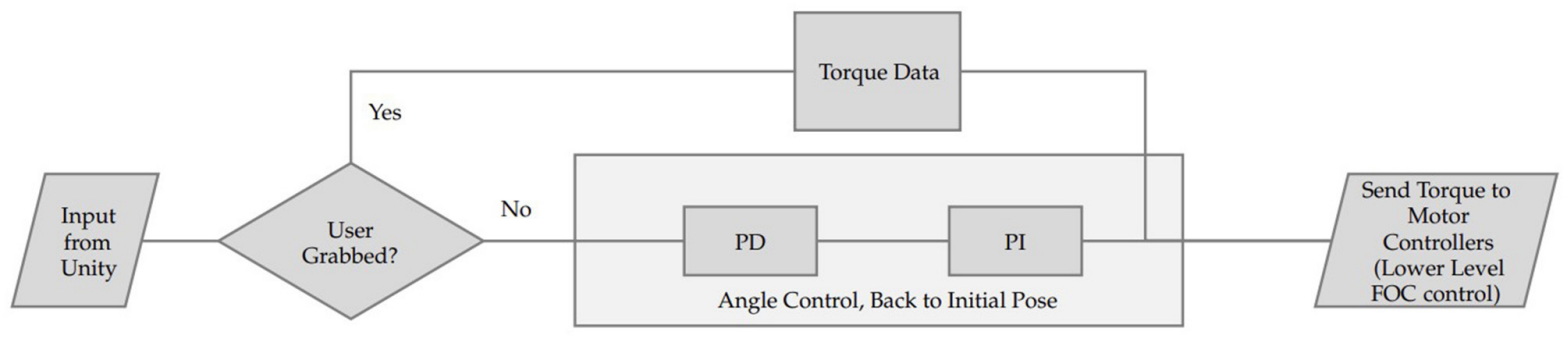
Haptic force rendering controller design.

Figure 8 shows the setup of the weight perception experiment. Upon completion of the experiment, the collected data was analyzed to determine the overall accuracy of weight perception. The findings were then used to pinpoint areas in which improvements to weight rendering should be focused.

#### 2.7.2. Assessing the effectiveness of force rendering

The aim of this research is to assess the effectiveness of force rendering. To evaluate this, we conducted an experiment designed to evaluate the accuracy of force direction rendering using Meta-Gravity. In this experiment, we presented a virtual ball to participants and asked them to grab it. We then applied forces with random directions (left, right, upper, lower, upper-right, upper-left, lower-left, and lower-right) to the ball, and participants had to identify the direction of the force for 10 trials. This allowed us to evaluate the effectiveness of the force rendering system in terms of direction accuracy. The experiment was conducted with 11 participants, and a total of 110 trials were completed.

**Figure 9:**
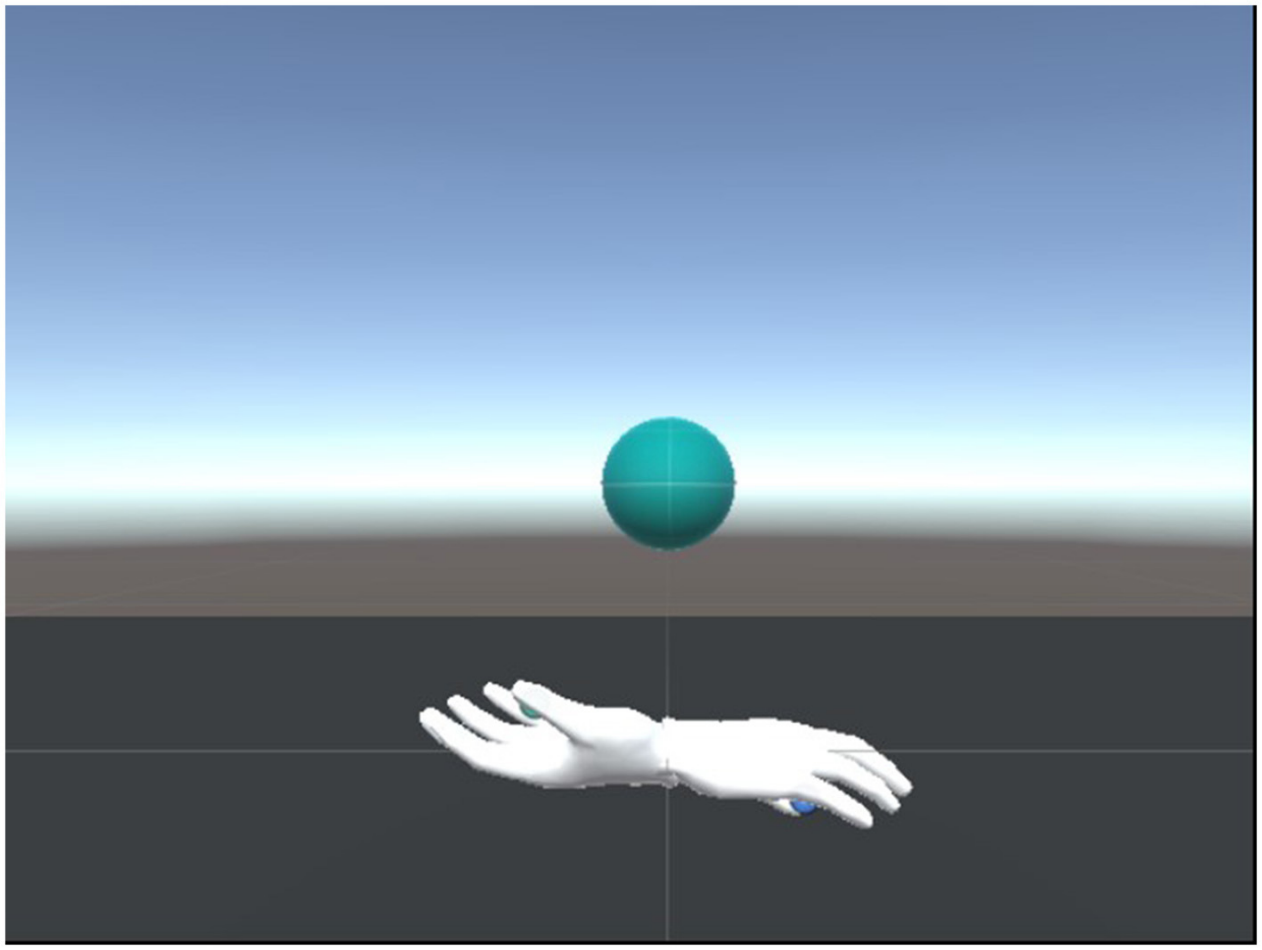
Participants were asked to grab the fixed ball, where forces with random directions are applied. They had to tell the perceived force direction.

Figure 9 shows the setup for the experiment. Participants were asked to grab the fixed ball, and forces with random directions were applied to it. Participants had to identify the direction of the force applied. This experiment provides a comprehensive evaluation of the effectiveness of force rendering in terms of direction accuracy.

#### 2.7.3. Manipulating various shapes with same weight

The effectiveness of shape rendering while maintaining a constant weight was evaluated using an experiment with a group of 10 participants. All of them were experienced with haptic devices and had no known motor impairments. The haptic device used in the experiment was designed to measure the force exerted by the participants and accurately simulate the shape of various objects. Participants were presented with objects of different shapes and sizes, all with the same weight. These included a sphere, a rectangle, a cup, a cone, and two cylinders with different radii and heights.

Participants wore a VR headset to observe the various shapes, without any physical information about the object, such as circumference or height. After a brief introduction to the haptic device and the experiment, as well as a training session to familiarize participants with the device, they were asked to grasp each object in a randomized order. Each object was explored for 30 s as participants attempted to describe its shape and estimate its size.

Participants rated the perceived realism of the haptic device in rendering the different shapes on a 5-point scale. In a separate session, they provided their predicted physical properties of the objects at a millimeter level.

The accuracy of the shape rendering was quantified by calculating the mean error between participants' descriptions of the objects and their actual properties. This combined subjective and objective elements. The subjective element was the participants' descriptions of their perception of the object's shape. The objective element was the participants' estimations of the object's dimensions.

This mean error served as a numerical representation of the accuracy of participants' perceptions, minimizing potential subjectivity associated with the task. Additionally, the subjective descriptions provided valuable qualitative insights into the efficacy of the haptic device in rendering different shapes. By examining these descriptions, potential patterns or recurring themes were identified, shedding light on the strengths and weaknesses of the device, and guiding further improvements.

**Figure 10:**
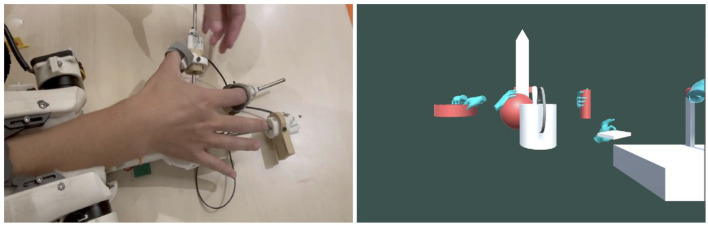
Testing for grabbing virtual objects.

Figure 10 demonstrates the scene for the shape rendering test, where participants can grab virtual objects of different shapes through the haptic device. A detailed demonstration of the function can be seen in our demo video (Chen, 2022). The evaluation of the experiment can provide insights into the accuracy of shape rendering through a haptic device. This experiment aims to understand how well the haptic device can replicate the properties of different shaped objects with the same weight and how accurately participants perceive these properties through the device.

## 3. Result

This section provides an overview of the experimental results, detailing the outcomes in three areas: perception of weight, perception of force direction and torque effectiveness, and shape rendering. Each subsection commences with an overview of the experimental setup, followed by a discussion of the results, and a visual depiction of the findings.

### 3.1. Perception of weight

This experiment involved 15 participants who were tasked with perceiving and sorting three balls of different weights. The primary objective was to evaluate their accuracy in ranking the balls by mass and to investigate the potential correlation between rendering accuracy and the weight of the objects.

**Figure 11:**
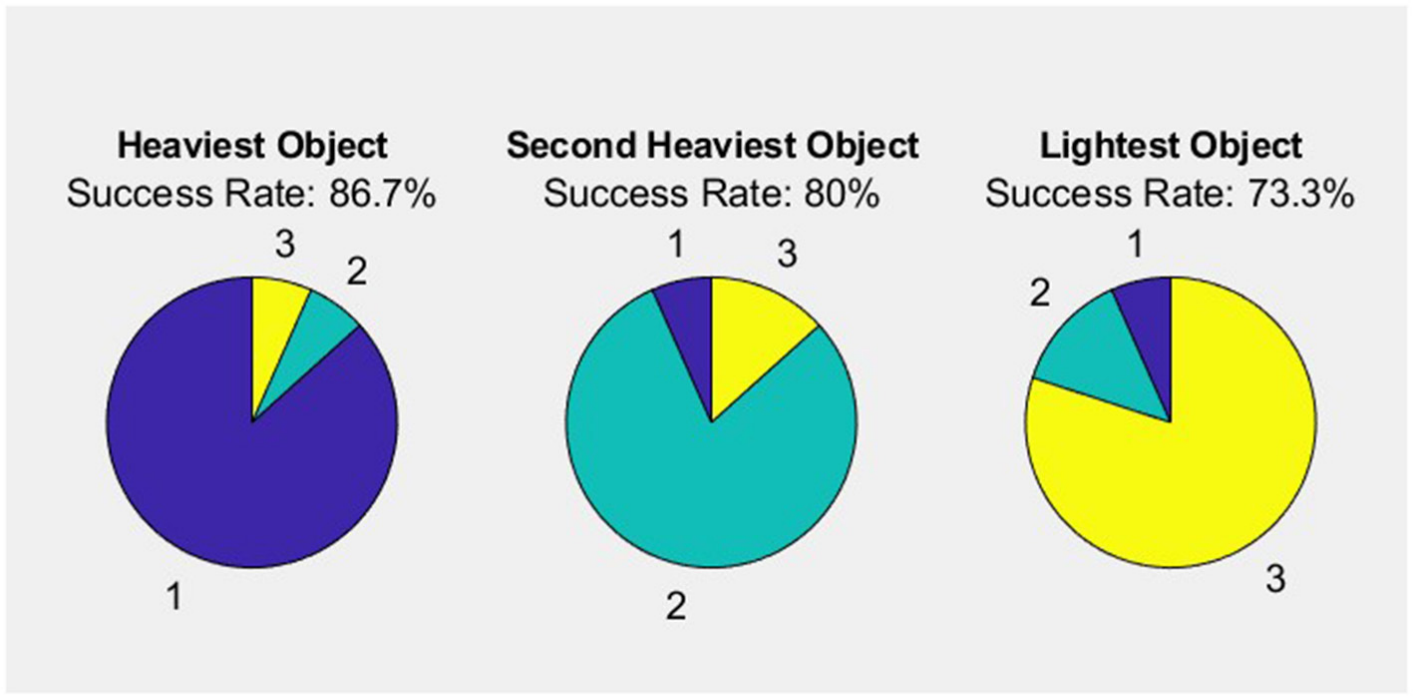
Evaluated weight ranking and accuracy.

Figure 11 demonstrates the results of the experiment, with three balls labeled 1, 2, and 3 and chosen by each person as Heaviest Object, Second Heaviest Object, and Lightest Object, respectively. The participants exhibited varying levels of accuracy in ranking the balls by mass: the heaviest ball was correctly identified in 13 out of 15 trials (86.7% accuracy), the medium ball in 12 out of 15 trials (80.0% accuracy), and the lightest ball in 11 out of 15 trials (73.3% accuracy). In summary, 11 participants (73.3%) were successful in correctly ranking the balls by mass, while four participants (26.7%) were not. The results suggest a possible relationship between rendering accuracy and object weight, which warrants further investigation to better understand the underlying factors and potential strategies for enhancing weight perception across different weight categories.

### 3.2. Perception of force direction and torque effectiveness

**Figure 12:**
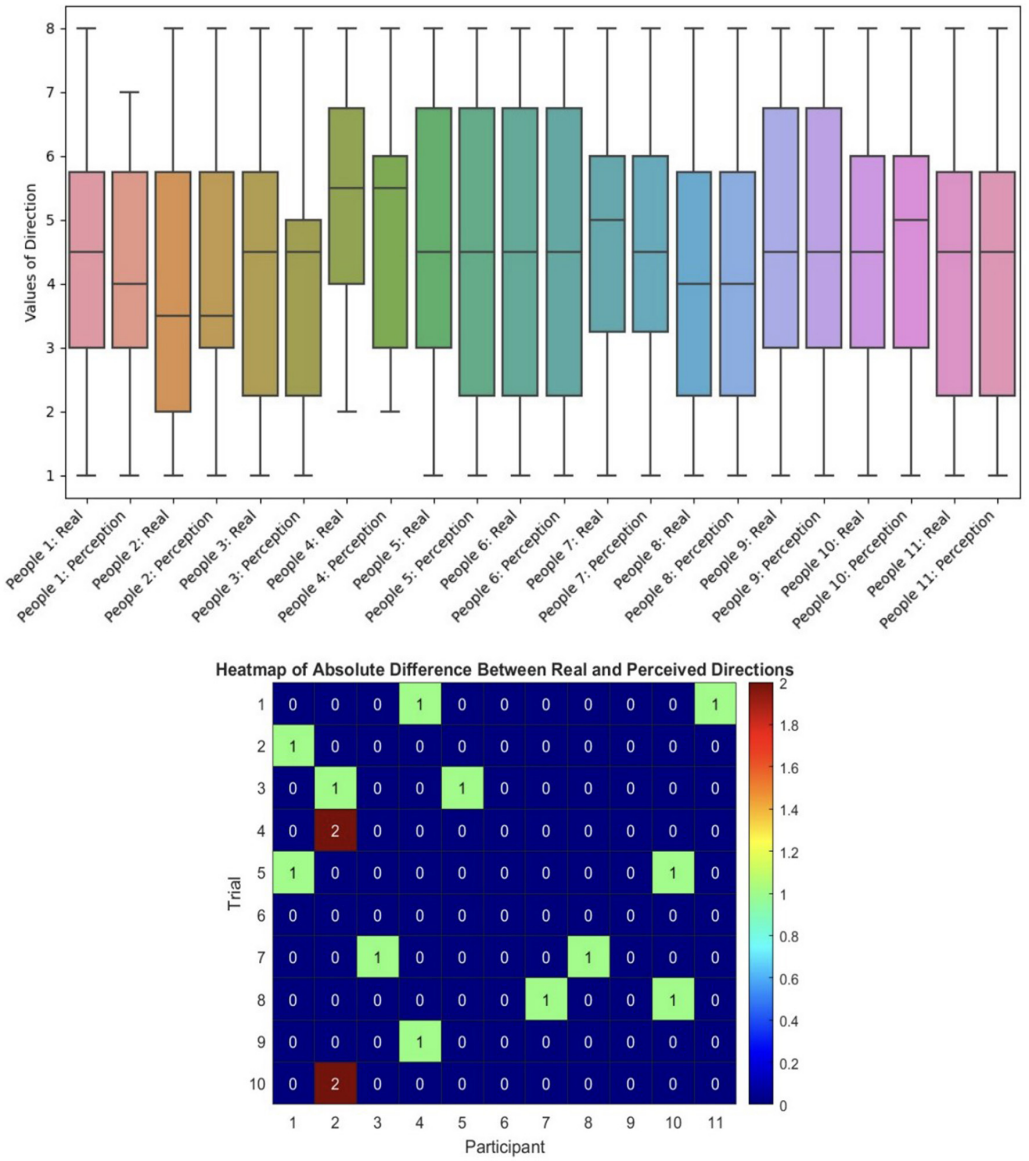
Human spatial perception compared to VR environment parameter and evaluation of the accuracy of human spatial perception.

This experiment is to test the force and its direction effectiveness, aimed at evaluating the accuracy of force direction rendering using Meta-Gravity, we conducted 10 trials on 11 participants each, resulting in a total of 110 trials. Eight random directions, including left, right, upper, lower, upper-right, upper-left, lower-left, and lower-right, were applied to the objects in the trials. Participants were not informed of the direction of each force after wearing the device initially, but they were aware that there were a total of eight different possible directions. They were given 5–10 s to determine the direction of the force, with the single force direction being completely random among the eight force choices. The Figure 12 shows the experiment data of 11 participates.

The Figure 12 presented in this paper illustrates a heatmap that visually represents the absolute differences between real and perceived directions. The heatmap serves as a tool to evaluate the accuracy of human spatial perception. A value of 0 indicates that an individual's perception aligns perfectly with the actual direction, thus demonstrating accurate spatial awareness.

The participants' verbally communicated force direction was compared to the actual direction of the force applied in the procedure. The overall correctness rate for the 110 trials was 87.3%, demonstrating that the accuracy of the direction rendering is good. Figure 12 displays the random sample data for these additional directions. These results suggest that the force rendering system performs well in terms of force direction accuracy, indicating its potential for various applications in haptic feedback and virtual reality.

### 3.3. Shape rendering result

**Figure 13:**
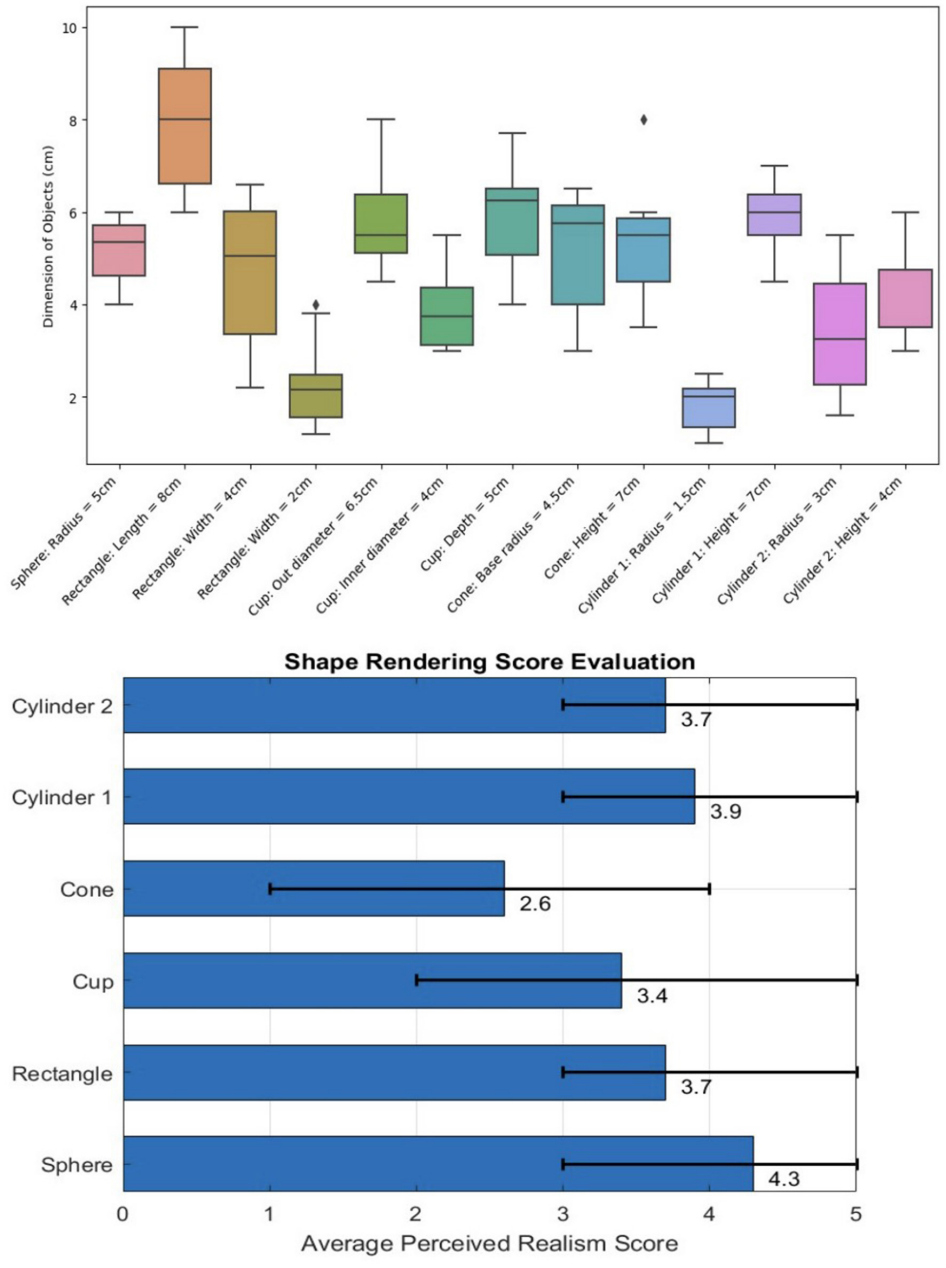
Comparison of participants' estimations for various object dimensions and shapes and evaluation of perceived realism score.

During the experiment, participants' descriptions of the objects, including estimated shape and size, were collected. The assessments for different shapes included: estimated radius of the sphere, estimated length, width, and height of the rectangle, estimated outer diameter, inner diameter, and depth of the cup, estimated radius and height of the base of the cone, and estimated radius and height of the base of both cylinders. The error was determined by calculating the absolute value of the difference between the object's scale set in the virtual environment and the mean value expected by the users. The Figure 13 shows the comparison of participants' estimations for various object dimensions and shapes. After analyzing the data collected, the error is calculated by accumulating the difference between each test value and the set value by each person and finally dividing it by the total number of people to get the result: Sphere: Set radius of 5 cm, radius error: 0.6 cm. Rectangle: Set dimensions of 8 x 4 x 2 cm, errors of 1.25, 1.45, and 0.71 cm, respectively. Cup: Set outer diameter of 6.5 cm, error 1.1 cm; inner diameter set at 4 cm, error 0.75 cm; depth 5 cm, error 1.2 cm. Cone: Set base radius of 4.5 cm, error 1.44 cm; height of 7 cm, error 1.9 cm. Cylinder 1 (smaller radius, larger height): Set radius of 1.5 cm, error 0.52 cm; height of 7 cm, error 1.15 cm. Cylinder 2 (larger radius, smaller height): Set radius of 3 cm, error 1.15 cm; height of 4 cm, error 0.9 cm.

Perceived realism was scored on a scale from 1 to 5, with 1 indicating “not real at all" and 5 indicating “very real." The average perceived realism ratings for each object were as follows: sphere: 4.3, rectangle: 3.7, cup: 3.4, cone: 2.6, cylinder 1: 3.9, and cylinder 2: 3.7. The Figure 13 shows the Perceived Realism Score and its distribution.

## 4. Discussion

The performance of our haptic device was evaluated through three primary experiments: perception of weight, perception of force direction and torque effectiveness, and shape rendering. This section will discuss these results and make comparisons to the performance of similar devices reported in the literature.

### 4.1. Weight perception accuracy and influencing factors

Subtitle: “Weight perception accuracy and influencing factors"

**Figure 14:**
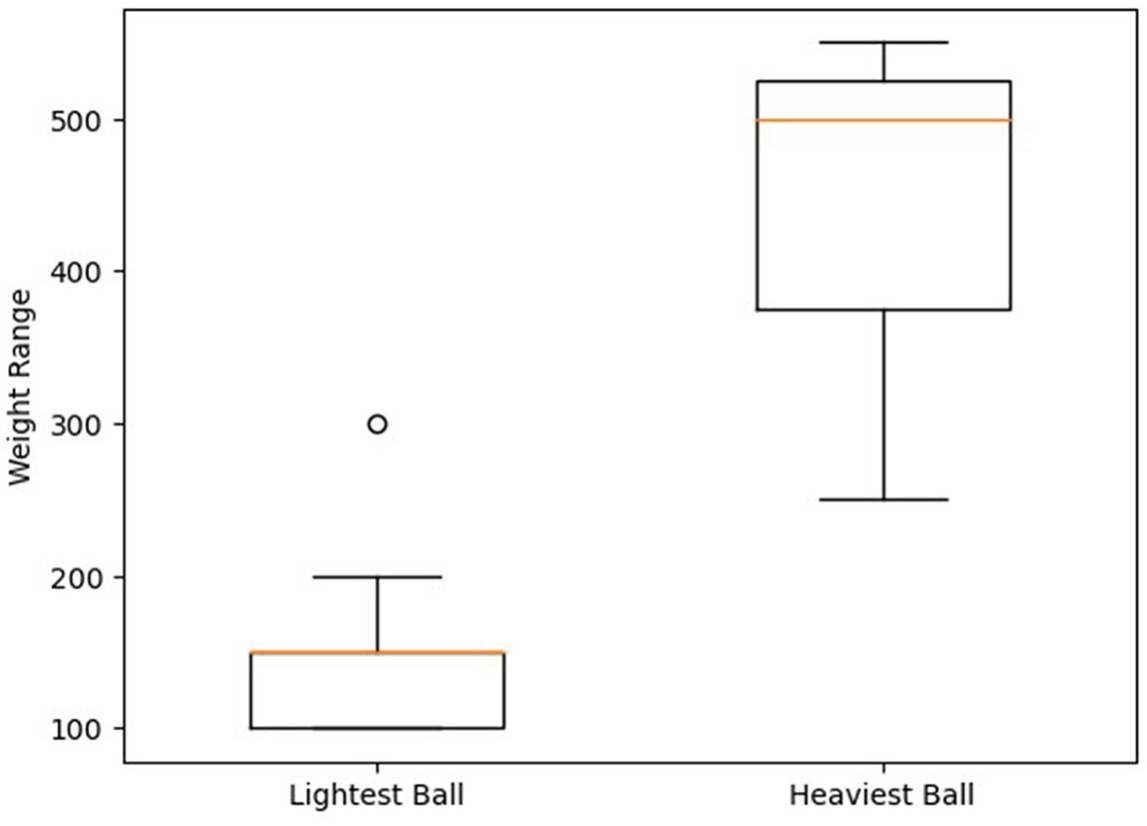
Estimated weights for the lightest and heaviest balls.

In the post-experiment questionnaire, participants were asked to estimate the approximate weight of the balls. Figure 14 illustrates that participants were generally able to accurately estimate the actual weight of the balls. The lighter ball (166.67 g) was often compared to real-world objects of similar weight, such as an apple, but was predominantly estimated to be lighter. This discrepancy could be attributed to natural human tendencies or a fatigue effect.

Estimations for the heavier ball were more precise, likely due to participants' familiarity with the weight of a 500 ml water bottle, which is equivalent to the rendered weight. During the study, two participants reported being disturbed by the reaction force exerted by the haptic device's fixed end on their forearm. This effect is believed to become more pronounced when larger forces are applied, suggesting that the impact of the haptic device on participant experience should be considered in future studies.

The weight perception experiment results revealed a trend suggesting a negative relationship between rendering accuracy and object weight. The accuracy was highest for the heaviest ball, slightly decreased for the medium ball, and was lowest for the lightest ball. This indicates that as the weight of the object decreases, participants' ability to accurately perceive and sort the balls by weight is diminished.

Several factors could have contributed to the observed trend in rendering accuracy concerning the weight of the objects, such as: Sensitivity: Participants might have been more sensitive to the differences in weight between heavier objects, allowing them to accurately perceive and sort the heavier balls with greater ease. Expectation: Participants may have had preconceived notions or expectations about the weight of the objects, which could have influenced their perception and decision-making process. Fatigue effect: Participants may have experienced a fatigue effect during the experiment, leading to the overestimation of the weight of lighter objects. Learning effect: It is also possible that participants became more adept at perceiving and ranking the balls as they gained more experience during the experiment, which could explain the increased accuracy for heavier balls.

Further research is needed to better understand the factors contributing to the observed relationship between rendering accuracy and object weight, and to explore ways to improve the accuracy of weight perception across all weight categories. Future studies should also consider the potential impact of the haptic device on participant experience and examine how this factor might be mitigated.

### 4.2. Exploring directional perception and individual differences in haptic feedback performance

In addition to the experiments, we conducted a brief evaluation of the haptic device's feedback latency, where our embedded system simultaneously reads the torque in real time and compares it to the ideal torque input. The difference and time delay between the two were employed to assess the effectiveness of the force presentation in terms of torque accuracy. As indicated by the bold plot, the motor system's response time is within 0.8 s. The results demonstrate that the force-presentation system is effective in terms of torque accuracy, exhibiting minor deviations and acceptable time delays.

The results of the experiment indicate that participants were generally successful in identifying the intended directions. However, some directions seemed to be more challenging for the participants to perceive accurately. For example, direction 7 (right) had a lower success rate compared to other directions, with an 11% discrepancy between the real and perceived values. On the other hand, directions 1 (upper) and 6 (lower right) had higher success rates with no discrepancies between the real and perceived values.

These findings suggest that certain directions might be more intuitive for participants to perceive, while others may require additional cues or practice to enhance accuracy. The results are in line with previous research on spatial perception, which has shown that people tend to perform better in tasks that involve perceiving horizontal or vertical directions compared to tasks that involve oblique directions (e.g., upper left, lower left, etc.; Gentaz et al., 2008).

One potential explanation for the observed differences in success rates could be related to the stimuli used in the study. It is possible that the visual or auditory cues for certain directions were more salient or distinguishable, leading to higher accuracy rates for those directions. Further research could investigate the impact of different sensory modalities on directional perception to provide better insights into the factors that contribute to successful direction perception.

Additionally, individual differences in spatial abilities and cognitive styles could also play a role in the observed variations in success rates across the directions. Future research could explore how individual characteristics, such as spatial intelligence, working memory, or attention, may influence the ability to accurately perceive and discriminate between different directions.

Moreover, the development of adaptive algorithms for force rendering may further enhance the system's performance by taking into account the user's individual perception of force and adapting the force rendering accordingly. This could be particularly beneficial in applications such as rehabilitation, training, and remote operation. Another aspect to consider is the integration of additional sensory feedback modalities, such as auditory or visual cues, to enrich the haptic experience. Combining multiple sensory inputs can lead to a more realistic and engaging interaction, potentially improving task performance and user satisfaction.

In conclusion, our findings contribute to the understanding of directional perception and highlight the importance of considering the role of individual differences and stimulus properties in designing tasks that involve spatial judgments. The high overall success rate indicates that participants generally performed well in perceiving the intended directions, but further research is needed to optimize the presentation of stimuli and task instructions to enhance accuracy in perceiving less intuitive directions.

### 4.3. Assessing haptic device performance and user perception across various object geometries in a virtual environment

The results of the experiment provide valuable insights into the performance of the haptic device and participants' ability to perceive and describe the shape and size of various objects in a virtual environment. Overall, the haptic device demonstrated a relatively good performance in rendering the objects' shapes, as reflected by the perceived realism scores. However, some objects were perceived more accurately than others, suggesting that the haptic device's performance might vary depending on the object's shape and complexity.

The sphere and cylinders yielded higher perceived realism ratings (4.3 for the sphere, 3.9 for cylinder 1, and 3.7 for cylinder 2), implying that the haptic device is more effective in rendering objects with simpler geometries. The lower scores for the cone (2.6) and cup (3.4) may indicate that the haptic device faces challenges in simulating objects with more complex shapes or a combination of curved and flat surfaces.

Regarding size estimation, the errors varied across objects, with the highest errors observed for the cone (1.44 cm for the base radius and 1.9 cm for the height) and rectangle (1.25, 1.45, and 0.71 cm). This discrepancy might be attributable to the participants' difficulty in perceiving the dimensions of these objects, the haptic device's limitations in rendering their shapes, or a combination of both factors.

It is crucial to consider potential improvements in the haptic device's design, functionality, or the experiment's protocol to enhance the accuracy of shape rendering and users' perception. Some possible avenues for further research and development include refining the haptic feedback mechanisms, improving the device's ability to render complex shapes, and providing more extensive training to participants on using the haptic device to reduce potential learning effects.

Additionally, investigating how factors such as participants' prior experience with haptic devices, spatial ability, or familiarity with the objects used in the experiment might influence the accuracy of their estimations and perceived realism scores would offer a deeper understanding of the factors contributing to the device's effectiveness and help guide future improvements in haptic technology.

In conclusion, the experiment demonstrated the potential of the haptic device in rendering virtual objects of varying shapes and sizes while maintaining consistent weight. The findings underscore areas for improvement and further investigation, paving the way for enhancing the performance of haptic devices and their applications in immersive virtual environments.

### 4.4. Comparison with haptic PIVOT device

To put our haptic device's performance into context, we compare our results with those from Robert Kovacs's study on Haptic PIVOT devices which is designed in 2020 (Kovacs et al., 2020). In Kovacs's study, participants correctly identified the heaviest ball in 83% of cases and the lightest ball in 91% of cases. The results of our study showed that participants correctly identified the heaviest ball in 86.7% of trials and the lightest ball in 73.3% of trials. Although our device's accuracy in identifying the heaviest ball is slightly higher than the Haptic PIVOT, our device has a lower accuracy in identifying the lightest ball.

Both studies found a tendency for participants to overestimate the weight of the balls. However, this effect may be more pronounced in our study, as participants predominantly estimated the lighter ball (166.67 g) to be lighter than its actual weight. In Kovacs's study, the lighter ball (90 g) was more accurately compared to real-world objects with similar weights, such as a tennis ball. Participants in both studies were generally able to accurately estimate the weights of the balls, with the heavier ball estimations being more precise in our study, likely due to participants' familiarity with the weight of a 500 ml water bottle.

In addition to the comparable accuracy in simulating forces, our haptic device offers several advantages over the Haptic PIVOT device. The improved mechanical structure and control logic using two motors enable our device to simulate forces in different directions and render rotational inertia, which may lead to a more immersive and realistic haptic experience. Furthermore, due to the difference in size and design, as well as the addition of a degree of freedom, our haptic device boasts a larger working space than the PIVOT, allowing it to accommodate a wider range of hand postures. This versatility makes our device more adaptable to various applications and user preferences. Lastly, our haptic device has the unique capability to simulate shapes, a feature that the PIVOT device lacks. This added functionality enhances the overall haptic feedback and opens up new possibilities for applications that require shape perception and manipulation.

These advantages, coupled with the promising results in terms of weight perception and rendering accuracy, demonstrate the potential of our haptic device as an effective and versatile tool for various applications in virtual reality and beyond. However, future research should continue to address the limitations observed in this study, particularly in rendering lighter objects, in order to further refine and optimize the performance of our haptic device.

### 4.5. Comparison with directional force feedback device

To contextualize the performance of our haptic device, we compare our results with those from Seonghoon Ban's study on Directional Force Feedback (DFF) devices in 2019, which are designed for immersive experiences in virtual reality (Ban and Hyun, 2019). In Ban's study, participants correctly identified the direction of force with an overall correctness rate of 83.8%. The results of our study showed that participants correctly identified the force direction with an overall correctness rate of 87.3%. Although our device's accuracy in identifying force direction is slightly higher than the DFF device, it is important to note that the experimental setups and participant samples may not be directly comparable.

Both studies aimed to evaluate the accuracy of force direction rendering in their respective devices. The slightly higher correctness rate in our study may be attributed to differences in the devices, the experimental setup, or the participant sample. Nonetheless, the results suggest that both devices perform well in terms of force direction accuracy, indicating potential for various applications in haptic feedback and virtual reality.

In addition to the comparable accuracy in rendering force directions, our haptic device offers several advantages over the DFF device. The improved mechanical structure and control logic enable our device to render forces in different directions and simulate rotational inertia, which may lead to a more immersive and realistic haptic experience. Furthermore, the heatmap visualization used in our experiment is an interesting way to display the results, as it clearly represents the absolute differences between real and perceived directions, providing a valuable tool for evaluating the accuracy of human spatial perception.

These advantages, along with the promising results in terms of force direction rendering accuracy, demonstrate the potential of our haptic device as an effective and versatile tool for various applications in virtual reality and beyond.

### 4.6. Comparison with visual-haptic size estimation study

In our shape rendering experiment, we compare our results with Nikolaos Katzakis's study on visual-haptic size estimation in peripersonal space (Katzakis et al., 2020). In Katzakis's study, participants estimated the size of spheres with different haptic references and gain levels, resulting in various degrees of discrepancy between the visual and haptic sizes. The psychometric analysis showed a central tendency effect, with participants overestimating the visual size for smaller haptic sizes and underestimating the visual size for larger haptic sizes. The just noticeable difference (JND) values for small, medium, and large haptic size references were 0.94, 1.12, and 1.15, respectively, indicating that the task difficulty was controlled well across the three tactile size references.

In our study, participants were asked to estimate the dimensions of various objects, including a sphere, rectangle, cup, cone, and two cylinders with different radii and heights. The errors in participants' estimations were calculated by comparing their estimated dimensions to the objects' dimensions set in the virtual environment. The comparison of participants' estimations for various object dimensions and shapes is shown in Figure 13.

The results of our study showed that participants were generally able to estimate the dimensions of the objects with varying degrees of accuracy. The perceived realism of our rendered objects was rated on a scale from 1 to 5, with 1 being “not real at all” and 5 being “very real.” The average perceived realism ratings for the sphere, rectangle, cup, cone, cylinder 1, and cylinder 2 were 4.3, 3.7, 3.4, 2.6, 3.9, and 3.7, respectively.

When comparing our results with Katzakis's study, it is important to note that while both studies involve visual-haptic size estimation, our study focuses on a wider range of shapes and dimensions. In addition, our study evaluates the perceived realism of the rendered objects, which is not assessed in Katzakis's study. Our haptic device's ability to simulate different shapes allows for a more versatile and immersive experience in virtual reality.

Despite the differences in the objects and tasks used in the two studies, both show the potential for further improvement in the accuracy and realism of visual-haptic size estimation. Future research should continue to refine the rendering techniques and explore the factors that influence size perception and discrimination in virtual environments, in order to enhance the overall haptic experience for users.

## 5. Future work and conclusion

This section rounds up the discussion, highlighting areas for future work that stem from the results of our experiments, drawing a conclusion based on the research, and identifying limitations to be addressed in further studies.

### 5.1. Future work

Building upon the findings and insights gained from this study, there are several directions for future research and development: Refining the haptic feedback mechanisms and improving the device's ability to render complex shapes, which could enhance user perception and performance in virtual environments. Investigating the role of individual differences, such as spatial ability, prior experience with haptic devices, and familiarity with the objects, to better understand the factors influencing user perception and performance. Developing adaptive algorithms for force rendering that take into account users' individual perception of force, which could be particularly beneficial in applications such as rehabilitation, training, and remote operation. Exploring the integration of additional sensory feedback modalities, such as auditory or visual cues, to enrich the haptic experience and create a more realistic and engaging interaction. Conducting more extensive training for participants on using the haptic device to minimize potential learning effects and enhance the accuracy of shape rendering and user perception.

### 5.2. Conclusion

This study has provided valuable insights into the performance of a haptic device and participants' ability to perceive and describe the shape, size, and weight of various objects in a virtual environment. The results demonstrated the potential of the haptic device in rendering virtual objects of varying shapes and sizes while maintaining consistent weight, and highlighted areas for improvement and further investigation. By addressing these limitations and building upon the findings, the performance of haptic devices and their applications in immersive virtual environments can be significantly enhanced. Overall, this research contributes to the understanding of user perception and interaction with haptic devices, and paves the way for future advancements in haptic technology.

### 5.3. Limitations

Despite the notable findings, this study is not without its limitations. The first is the relatively small sample size, which can affect the generalizability of the results. While we sought to include participants with varying levels of familiarity with haptic devices, a larger and more diverse sample could provide a more comprehensive understanding of user interaction with haptic technology.

Second, while our experiment primarily focused on shape rendering, it would be beneficial to investigate how the device performs when rendering more intricate characteristics such as texture and temperature, which are crucial aspects of tactile perception.

Third, this study concentrated on individual use of the haptic device, without considering scenarios where multiple users interact simultaneously in a shared virtual environment. Understanding how haptic devices perform under multi-user scenarios would be a valuable direction for future work.

Finally, the subjective nature of some parts of the evaluation-like descriptions of shape-could potentially introduce bias. Although steps were taken to mitigate this bias by having participants also estimate the physical properties of the objects, it remains a limitation to be considered.

## Data availability statement

The datasets presented in this study can be found in online repositories. The names of the repository/repositories and accession number(s) can be found in the article/supplementary material.

## Ethics statement

The studies involving human participants were reviewed and approved by Zhejiang University. The patients/participants provided their written informed consent to participate in this study.

## Author contributions

ZZ and CQ collaboratively conceptualized and constructed the system and device, carrying out an in-depth interpretation of the gathered data, and meticulously composed and revised the manuscript. ZZ took on the responsibility of data collection and analysis. All authors have diligently reviewed and unanimously approved the final manuscript.
